# Advanced fluorescence microscopy techniques for the life sciences

**DOI:** 10.21542/gcsp.2016.16

**Published:** 2016-06-30

**Authors:** John S. H. Danial, Yasmine Aguib, Magdi H. Yacoub

**Affiliations:** 1 Department of Chemistry, University of Oxford, Oxford OX1 3TA, United Kingdom; 2 Aswan Heart Centre, Aswan, Egypt; 3 Qatar Cardiovascular Research Centre, Doha, Qatar; 4 Harefield Heart Science Centre, National Heart and Lung Institute, Imperial College London, United Kingdom; 5 Present address: Max Planck Institute for Intelligent systems, Heisenbergstraße 3, 70569 Stuttgart, Germany

## Abstract

The development of super-resolved fluorescence microscopy, for which the Nobel Prize was awarded in 2014, has been a topic of interest to physicists and biologists alike. It is inevitable that numerous questions in biomedical research cannot be answered by means other than direct observation. In this review, advances to fluorescence microscopy are covered in a widely accessible fashion to facilitate its use in decisions related to its acquisition and utilization in biomedical research.

## 1. Introduction

The advent of optical microscopy has had a significant impact on the life sciences specially in visualizing the molecular architecture of the cell^1–11^. The development of fluorescent probes has allowed observations at the single molecule level^12^ whilst the recent introduction of super resolution microscopy has allowed imaging of an array of nanoscopic biological complexes with unprecedented resolution^1,5–7,9^. In this review, advanced fluorescence microscopy techniques are discussed in an application-oriented fashion.

## 2. Fluorescence

A quantum mechanical treatment of an atom reveals the probability of an associated electron, possessing an amount of available energy, of occupying any energy level of the atom. At scales larger than molecular scales, levels group into bands, sometimes referred to as *states*. Often, electrons occupy the lowest energy state (referred to as the *ground state*). Electrons could also occupy higher energy states if the ground state is fully occupied or if they are provided with the energy differential sufficient to *excite* them from the ground state to any unoccupied upper energy state. The energy differential can be provided in the form of light. Light is composed of quantized elementary particles known as *photons*. It is now established, following years of experimentation, that photons possess two natures, a wave nature and a particle nature^13^. Besides mass and speed, which reflect their particle nature, photons are also characterized with wavelength and frequency, which reflect their wave nature. Wavelength describes how often a wave repeats itself on the length scale, and frequency, describes how often a wave repeats itself but on the time scale. The energy of a photon is directly proportional to its frequency and inversely proportional to its wavelength.

**Figure 1.**
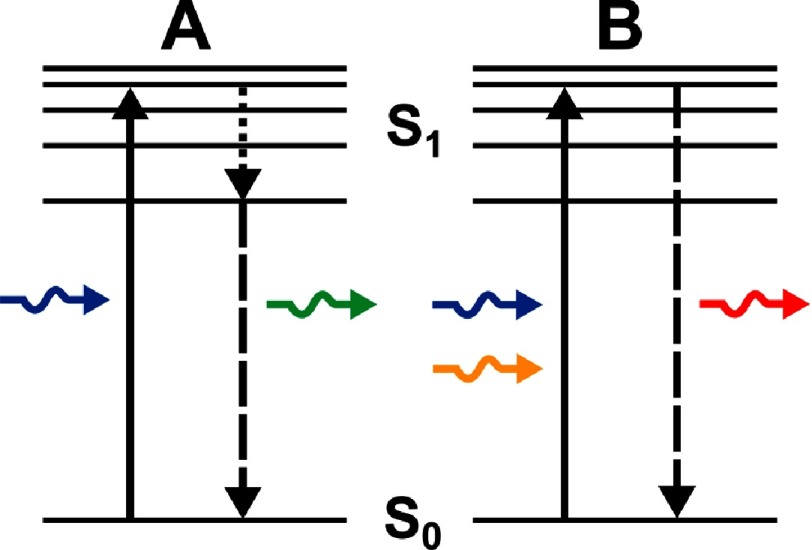
Schematic diagram representing energy levels in an atom. (A) Spontaneous emission: an electron excited (solid black arrow) by an incident photon (blue arrow) from the ground state (S_0_) to the first state (S_1_) spontaneously relaxes by internal conversion (dotted line) to the lowest energy level of S_1_ and then to S_0_ by emitting another photon (green arrow) of higher wavelength. (B) Stimulated emission: an electron excited (solid black arrow) by an incident photon (blue arrow) from the ground state (S_0_) to the first state (S_1_) can be stimulated to relax to S_0_ by another photon (orange arrow) of higher wavelength and emitting a photon (red arrow) of higher wavelength. The wavelength of a spontaneously emitted photon is higher than that produced by stimulated emission.

A coherent light source (e.g. laser) emitting photons with a known wavelength can be used to excite an electron in an atom, molecule or bulk, to an energy state higher than the ground state provided that the energy difference is less than the energy of the excitation photon. Following excitation, electrons could spontaneously, or be stimulated to, relax to the ground state. Either result in the emission of a photon of different energy, and thus different wavelength, to the excitation photon. This process is known as *fluorescence*. The wavelength of a spontaneously emitted photon is higher than that emitted stimulatingly. These processes can be figuratively depicted using a simplified Jablonski diagram (Figure 1). Emission at different wavelengths can be split using a *dichroic* mirror; a mirror that has different transmission properties at different wavelengths. This implies that the excitation and emission can be split; the latter to be collected and the former to be blocked.

## 3. Fluorescent labels

Observation of single biological molecules is far beyond the realm of the naked eye and white light microscopy. To facilitate observation at the single molecule level^14^, a fluorescent molecule, referred to as a *label* or a *fluorophore*, is attached to the biological molecule of interest, excited using a coherent light source and the emitted fluorescence is, consequently, collected. Improved camera technologies and fluorescent labels have rendered this possible.

The decision to choose an appropriate fluorescent label relies on three important parameters: photo stability, brightness and size. Following excitation, an electron could relax to the ground state, and emit a photon, or relax to a non-emissive state and not emit. The latter process is known as *photoblinking*. Relaxation to the non-emissive state is followed by relaxation to the ground state and, subsequently, resumed ability to fluoresce after excitation. It is evident that this process is temporary and thus the term blinking. *Photobleaching*, or permanent loss of fluorescence, can occur due to an alteration to the chemical structure of a fluorescent label following long periods of laser exposure. Typically, photo blinking occurs on the scale of a few seconds, whilst photobleaching occurs after 0.1 –1000 seconds of continuous exposure depending on the label.

Although often implicated as a limiting factor in single particle tracking experiments, photobleaching has been useful in determining the stoichiometry of protein complexes. It was shown that by exciting singly-labeled monomers in an oligomeric complex and recording the overall decrease in intensity caused by photobleaching, the stoichiometry of the complex under study can be deduced by counting the photobleaching steps^15^.

**Figure 2.**
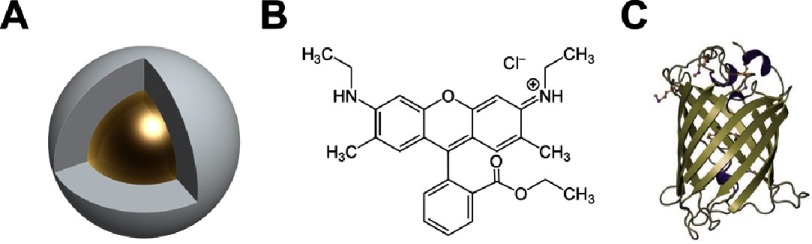
Schematic diagram representing different fluorescent labels. (A) Quantum dots are inorganic semi-conducting nanocrystals composed of a core (e.g. CdTe, CdSe, InP, InGaP etc...) and a shell (e.g. ZnS). The shell can be functionalized (i.e. grafted) with a chemical moiety to enable targeted labeling of a protein. Furthermore, the size and composition of the shell can be tuned to improve the photo stability of a quantum dot for single molecule imaging purposes. Though possessing high quantum yields, quantum dots suffer from poor intercellular internalization and uncharacterized size-dependent effects on protein dynamics. (B) Organic dyes are small fluorescent moieties that can be linked to the protein under study via a chemical tag. The small size and high quantum yield of organic dyes render them suitable labels for single molecule imaging. However, their potential phototoxic effects make a large subset, yet, unsuitable for imaging in living cells. Shown in the figure is the chemical structure of the organic dye Rhodamine 6G. (C) Fluorescent proteins are genetically expressible labels which are suitable for imaging in living systems. However, their utility for single molecule imaging is dependent on their proper folding and the size of the protein under study. Shown in the figure is the structure of the Green Fluorescent Protein (GFP). Figure not to scale.

In general, photostability (i.e. photo - blinking and bleaching) determine the fate of a fluorescent label and can sometimes limit its application in biophysical investigations. Whilst photo stability determines the traceability of a fluorescent label, brightness determines its detectability. *Quantum yield* or the ratio of the number of photons emitted to the number of photons received by a fluorescent label. Higher quantum yields denote higher probabilities of fluorescence detection. There is a limit, however, on how bright a label could be. A large label is typically brighter than a smaller label. Yet, for many biological processes, where the molecule of interest is smaller than the label or its dynamics can be affected by the size of the label, a large label cannot be used (Figure 2). Other parameters such as labeling schemes, coupling efficiency, and compatibility with live-cell imaging are also considered when choosing an appropriate label (Table 1). A detailed overview of the different labels and their associated parameters can be found in [16–18].

**Table 1:** Comparison between different fluorescent labels.

Fluorescent label	Size / structure	Photo bleaching / blinking times	Brightness	Labeling methodologies	Reported applications^*^	Available labels^*^
**Quantum Dot (QD)**	6 –60 nm / Inorganic nanocrystal	>1000 s / <1 ms^67^	200 – 2000 mM.cm^−1^^68^	Bio conjugation, or electrostatic interaction^69^	Tracking of single membrane and motor proteins *in vitro and in vivo*^70^	CdTe, CdSe, InP, InGaP, PbS, PbSe, and ZnS-coated CdSe^16^
**Organic dye**	0.5 nm / organic molecule	1 – 10 s / 0.5 s^71^	45 – 250 mM.cm^−1^ ^16^	Targeted labeling via a peptide tag (e.g. FLAG-tag, HA-tag or His-tag), or, a protein tag (e.g. Halo-tag)	Super resolution imaging of histone proteins *in vivo*^72^. Tracking of membrane and motor proteins *in vitro*	For a list of organic dyes refer to [73]
**Fluorescent protein (FP)**	3 nm / protein	13 – 174 s / 1 -2 s^18^	4 – 95 mM. cm^−1^ ^18^	Protein expression or targeted labeling via a peptide or protein tag.	Imaging the dynamics of filopodia, microtubules, transcription factors, chromosomes, myosin, and, cadherin *in vivo*^33^	For a list of fluorescent proteins refer to [18]

**Notes.**

* Non-exhaustive

## 4. Fluorescence microscopy techniques

### 4.1. Diffraction-limited microscopy techniques

**Figure 3.**
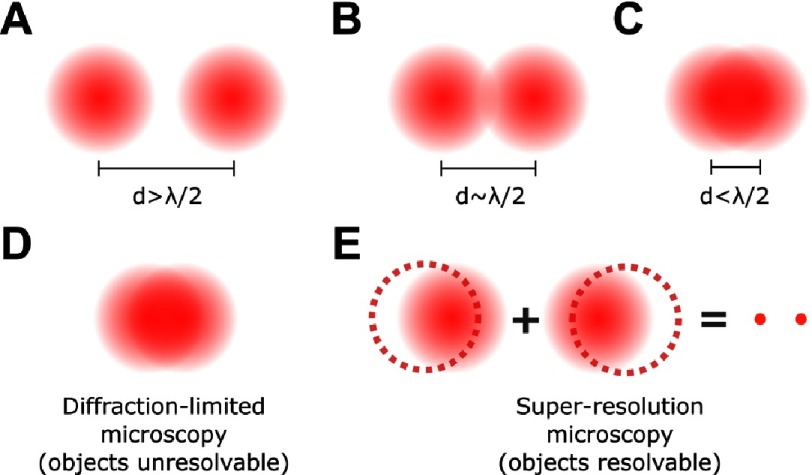
Schematic diagrams representing the diffraction limit of light. Due to the wave nature of light, a point object observed in the far field is seen as an airy disk. If the distance between two point objects is (A) greater than, or, (B) equal to half of wavelength of the excitation source, the objects become resolvable, and can be precisely located. If the distance between the two point objects is (C) less than half of the wavelength of the excitation source, the objects become unresolvable. This resolution limit is known as the Abbe diffraction limit. Closely located point objects which are not resolvable using diffraction-limited microscopy techniques (D) can be resolved using super-resolution microscopy techniques (E). Unlike diffraction-limited microscopy techniques which operate by detecting the fluorescence from all the molecules in a single image, super-resolution microscopy detects the fluorescence from each molecule, or a subset of molecules, at different times; overlapping the images obtained for distinct fluorophores results in a super-resolved image.

The wave nature of light imposes a fundamental limit on the dimensions of the smallest object that can be observed using light microscopy. The limit was derived by Ernest Abbe who found that any two objects could be resolved if the distance in between is approximately larger than half of the wavelength of the excitation source. This implies that any two fluorescently-labeled entities, observed using visible light (450 –650 nm), cannot be discerned if they are closer than 200 –300 nm (Figure 3). Fluorescence microscopy techniques which cannot overcome that resolution (diffraction) limit are known as diffraction-limited and are discussed below. Super resolution microscopy techniques which can overcome the diffraction limit of light are explained in the next section.

#### 4.1.1 Epi-fluorescence illumination microscopy

**Figure 4.**
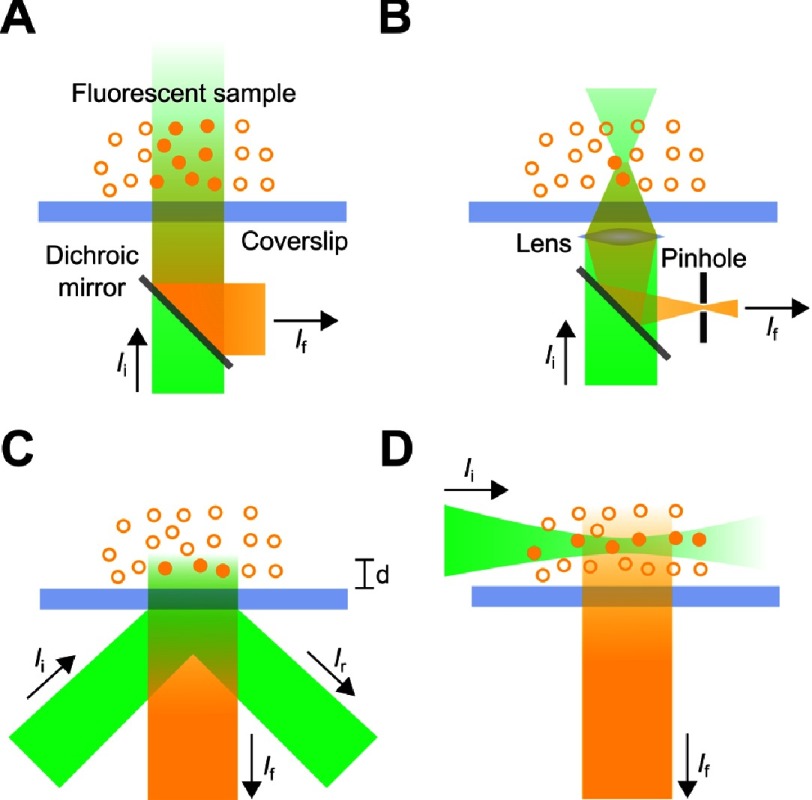
Schematic diagrams representing various diffraction-limited fluorescence microscopy techniques. In Epi-illumination (A), an upwardly-directed laser beam, under filling a lens objective, is directed perpendicular to the plane of the fluorescent sample and the fluorescence, from a large portion of the sample, is collected using the same objective. For large samples, the acquired image is blurred as a result of the overlapping of the fluorescence signal obtained from the different imaging planes. In confocal microscopy (B), an upwardly-directed laser beam is focused at specific planes of a sample to excite fluorophores inside the focusing spot. By scanning the beam laterally across the sample, a full image of a specific plane can be acquired. Acquisition rates in confocal microscopy are limited by the scanning speed of the beam rendering image acquisition slow. To resolve that problem, light is directed at an angle, above the critical angle, to undergo total internal reflection at the interface (C). To satisfy the boundary conditions at the interface, an evanescent (decaying) wave propagates for a few hundred nanometers above the boundary exciting fluorophores at the vicinity of the interface. The acquired image is not blurred as a result of the emission being limited to a thin imaging plane. Furthermore, acquisition rates in TIRF microscopy is only limited by the camera frame rate rendering image acquisition fast. In light sheet microscopy (D) a laser beam is directed though a cylindrical objective, or a two-dimensional photonic lattice, creating a light sheet that excites a section through a fluorescent sample. The fluorescence from the excited plane is orthogonally collected. LSFM combines fine optical-sectioning with fast acquisition rates. I_i_ is the incident beam, I_r_ is the reflected beam, and, I_f_ is the fluorescence emission.

The use of epi-illumination had originally referred to the arrangement of optical components that permits illumination from above the specimen. The term now refers to any orthogonal scheme of illumination whereby the excitation source is directed perpendicular to the plane of the specimen. Epi-fluorescence illumination has, and still is, implemented using xenon arc & mercury vapor lamps, Tungsten-Halogen, and Light Emitting Diodes (LEDs)^19^. However, the strong divergence and incoherence of these excitation sources meant that single-molecules could hardly be observed in thin samples even at substantially-high light levels. To observe single molecules using epi-fluorescence illumination, a laser beam is directed through an objective lens onto a sample and the fluorescence emission is collected using the same objective^12,20^. The emission and excitation are separated, where then, the former can be imaged (Figure 4A). Although this scheme serves to effectively image thin (<1 µm) samples, it fails to image dense thick samples where the fluorescence signals from the different imaging planes destructively contribute to the acquired image. The simple illumination scheme has served to uncover the reversible assembly of the ‘Tat A’ component of the twin-arginine protein transport system^21^.

#### 4.1.2 Confocal microscopy

To ameliorate the aforementioned problem, an upwardly-directed laser beam is focused inside the sample and a micron-sized pinhole is placed in the emission path to restrict the emission to that from a micron-sized spot and eliminate any out-of-focus signal (Figure 4B). To image a single plane, the spot is laterally scanned across the sample^22^. Scanning is typically performed by an electrically-expandable material, a set of electronically-controlled mirrors or a multiple-hole spinning disk which deflect the focused beam laterally across the sample. In all cases, acquisition is not set by the detector’s frame rate, as in epi-illumination microscopy, but by the scanning speed. Although the fine sectioning of biological samples in confocal microscopy produces high-resolution images, the limitation on the acquisition speed renders confocal microscopy an unsuitable technique for imaging highly dynamic processes.

#### 4.1.3 Total Internal Reflection Fluorescence Microscopy (TIRF-M)

Light incident at a boundary between two different media changes its direction of propagation; this process is known as *refraction*. If the angle of incidence exceeds the *critical angle* dictated by the ratio of the refractive indices of the two mediums, light undergoes *total internal reflection*. To satisfy boundary conditions, an evanescent (decaying) field propagates parallel to the plane of the boundary. The depth of penetration of the evanescent field is dictated by the angle of incidence and the ratio of the medium’s refractive indices; however, it can be adjusted to a few hundred nanometers. The evanescent field exclusively excites fluorophores at the vicinity of the boundary. This illumination scheme is known as Total Internal Reflection Fluorescence Microscopy (TIRF-M) (Figure 4C)^23^.

**Figure 5.**
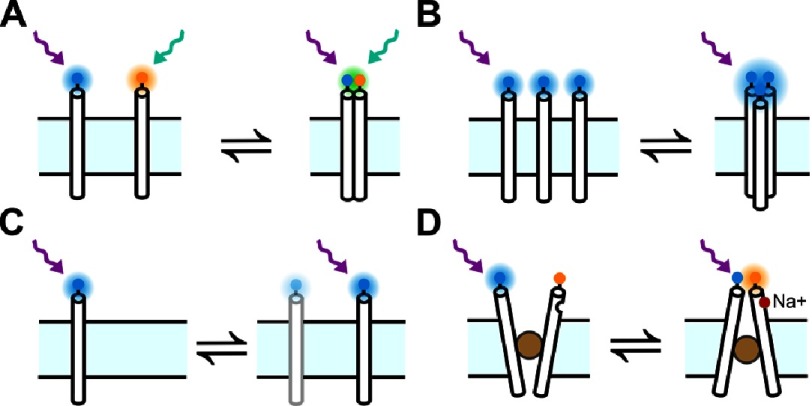
Schematic diagrams representing various single molecule techniques used in studying the function and dynamics of membrane proteins. (A) The binding of two different fluorescently-labeled subunits can be detected by the presence of transient co-localized fluorescence bursts in two emissions channels. (B) The clustering of multiple subunits can be detected observing an increase in fluorescence. The number of subunits in a single cluster can be deduced by dividing the fluorescence signal of the cluster by the fluorescence signal of a single subunit. Alternatively, a time sequence of the cluster is acquired as it photobleaches and the number of subunits can be deduced by counting the photobleaching steps. (C) The random movement (diffusion) of a protein can be imaged and the hydrodynamic properties of the membrane, and surrounding, can be obtained from the calculated diffusion coefficient. (D) The gating and alternating action of an ion channel, or transporter, can also be detected by single-molecule FRET. In FRET, energy is transferred between a donor (blue) and acceptor (orange) when they are less than 10 nm apart. The ratio of the emission of the acceptor to the emission of the donor, known as the FRET ratio, is a direct measure of how far both fluorophores are. By labeling ion channels, and other biomolecules, at closely located sites, using suitable FRET pairs, and monitoring the FRET ratio, it is possible to observe proteins dynamics in real time. Reproduced with permission from [63].

**Figure 6.**
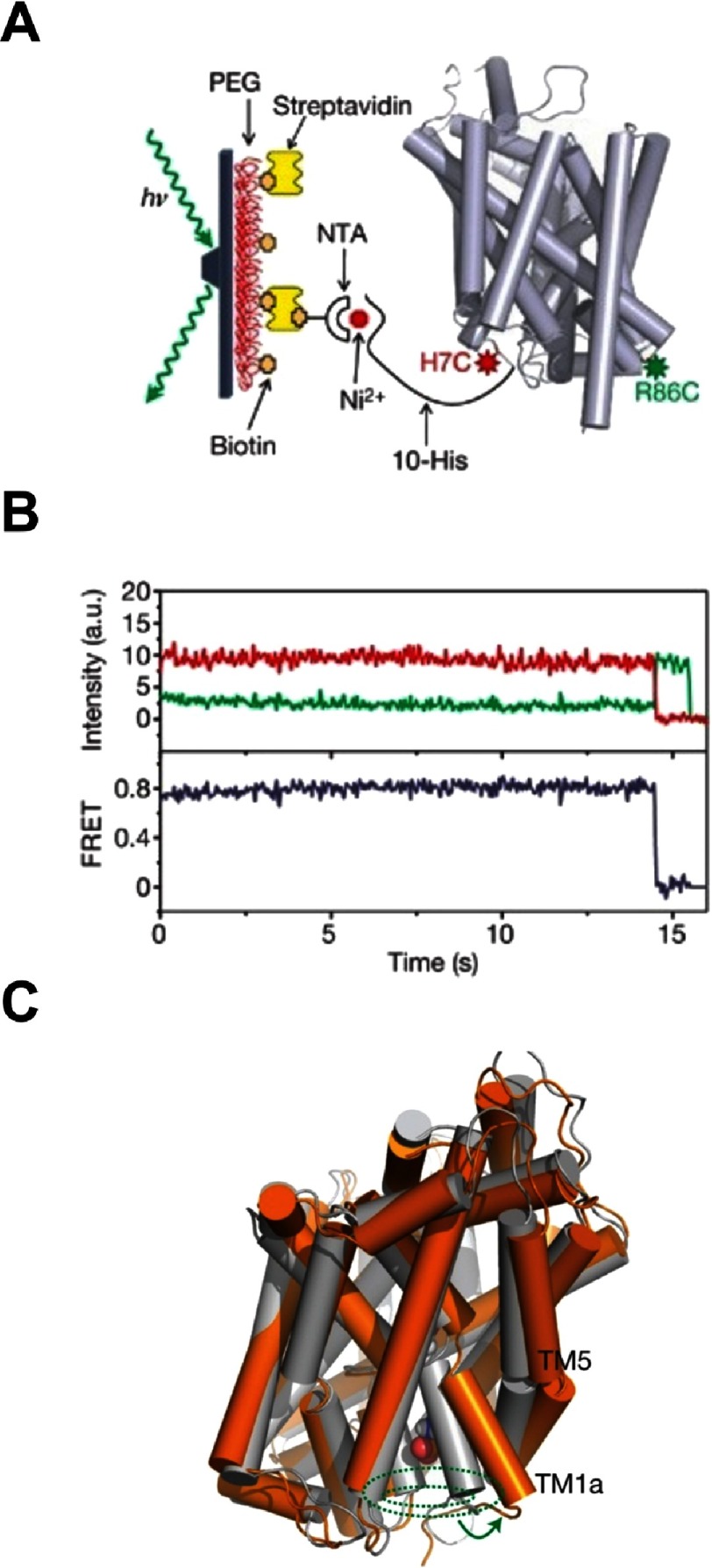
Reported example for the use of single molecule FRET in imaging the gating dynamics of the neurotransmitter transporter homologue (LeuT). (A) The diffusion of doubly-labeled LeuT is restricted through binding to a glass substrate. A laser beam undergoes total internal reflection at the glass / water interface and the evanescent field excites the fluorescently labeled protein. (B) The emission from the donor and acceptor dyes is recorded and the FRET ratio, defined as the ratio of the acceptor-to-donor emission, is calculated under different physiological conditions. (C) The observed dynamic changes can be translated into their structural context with the aid of molecular dynamics simulations. Reproduced with permission from [64].

Due to its simple implementation, TIRF-M found numerous applications in biology, especially where it relates to imaging lipid membranes and its constituents. The superior signal-to-noise ratio of TIRF-M has been configured for real time measurements on numerous transporters and channels at the single molecule level^24–28, 29–31^ (Figures 5 and 6). Although lipid membranes and their constituents can be easily imaged in TIRF-M, the richness of intra-cellular biology is not accessible to this technique.

#### 4.1.4 Light Sheet Fluorescence Microscopy (LSFM)

Light sheet microscopy was developed to overcome the limitations imposed by confocal and TIRF microscopies^32^. Light sheet microscopy relies on an orthogonal illumination scheme whereby a couple of microns’ thick sheet of light, produced by directing a Gaussian-shaped beam though a cylindrical lens objective, illuminates a section of a fluorescently labeled sample. The fluorescence signal from the section is collected by another objective placed perpendicular to the excitation beam (Figure 4D).

**Figure 7.**
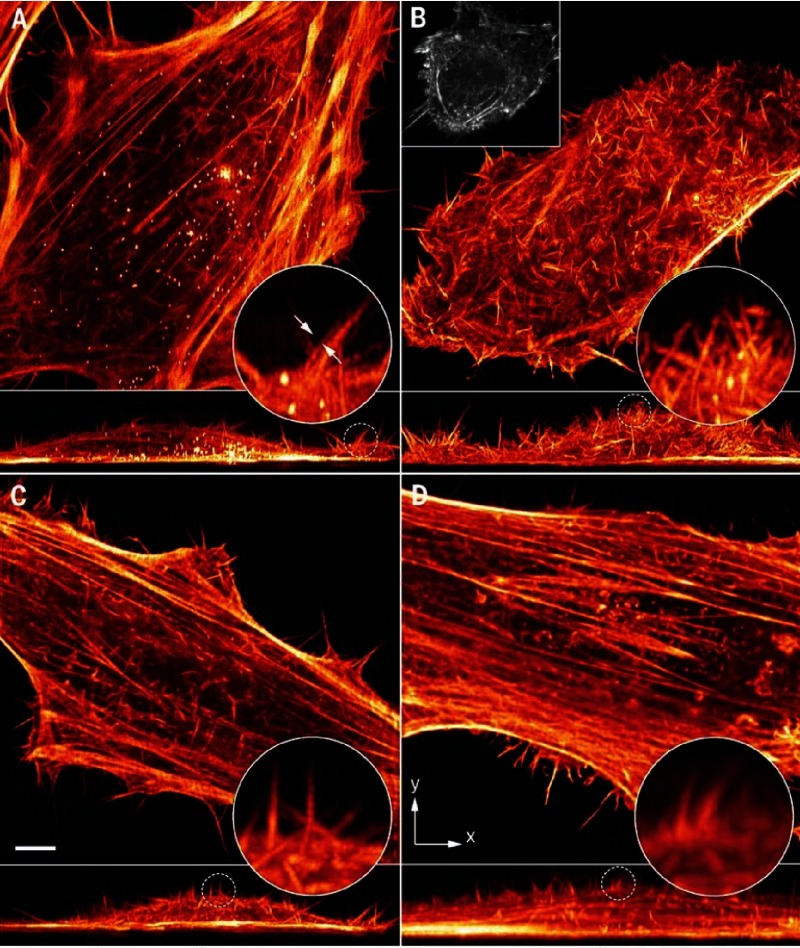
Hela cells labeled with the fluorescent protein mEmerald imaged using lattice light sheet microscopy in different configurations. Scale bar = 5 µm. Reproduced with permission from [33].

Although this scheme enables video rate imaging and effectively reduces photo bleaching, the thickness of the light sheet does not permit fine sectioning, and, therefore images suffer from reduced contrast. Lattice light sheet microscopy was recently reported by Chen and coworkers where the cylindrical-lens system was replaced by a two-dimensional optical lattice^33^. This configuration produces a hundred nanometers’ ultra-thin light sheets that can be used to illuminate thin sections of a sample. This improvement permits 3D intra-cellular imaging at sub second temporal resolutions combined with unprecedented axial and lateral spatial resolutions (Figure 7). Light sheet microscopy has been instrumental in uncovering the organization of RNA polymerase II *in vivo*^10^, tracking single molecules on the apical membrane of living cells^34^ and imaging mouse embryos during the first development stages^35^.

### 4.2 Super resolution microscopy techniques

**Figure 8.**
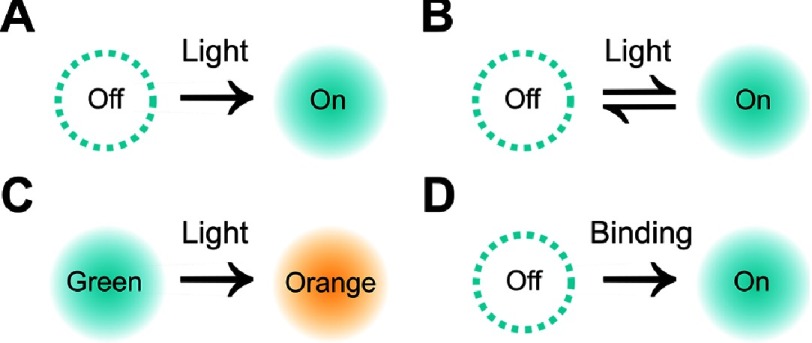
Schematic representation of some fluorescence modulation schemes. (A) Photoactivation: a deactivated (switched off) protein is irreversibly activated (switched on) by the application of light. (B) Photoswitching: a deactivated protein is reversibly activated by the application of light. (C) Stimulated emission: an active protein can spontaneously emit (green) or be stimulated to emit (orange). (D) Binding-induced activation: a deactivated (switched off) protein is irreversibly activated (switched on) upon binding to a substrate. In a fluorescent sample, the fluorescence of some fluorophores is sequentially modulated discriminating them from other fluorophores whose fluorescence has not been modulated. Successive modulations result in obtaining a super resolved image where the fluorescence from single molecules is well resolved.

Since many biological molecules perform their functions cooperatively, in nanoscopic molecular complexes (5 nm –100 nm), diffraction-limited fluorescence microscopy techniques fail to uncover the architecture, and eventually dynamics, of those biological machineries. To overcome the diffraction limit, a set of fluorescence microscopy techniques, known as super resolution microscopies, were developed since 1994. Super resolution microscopies rely on localizing individual fluorophores by sequentially modulating their fluorescence and constructing a super-resolved image by super position of the exclusively localized fluorophores. Methods for fluorescence modulation are figuratively summarized in Figure 8 and explained in [36].

#### 4.2.1 Photoactivated Localization Microscopy (PALM)

The engineering of photoactivatable fluorescent proteins has prompted the development of PALM in 2006^37^. In PALM, a weak short-wavelength laser beam randomly activates a subset of inactive fluorophores. The activation beam has a low intensity enough to ensure that fluorophores in the same diffraction limited region are unlikely to be all activated at the same time. Another beam, of longer wavelength and higher intensity, is used to excite the activated fluorophores and the emission is subsequently collected. Since the distance between the majorities of the active fluorophore pairs is larger than the diffraction limit, the centers of the associated airy disks can be determined and the position of the fluorophores can be precisely located.

**Figure 9.**
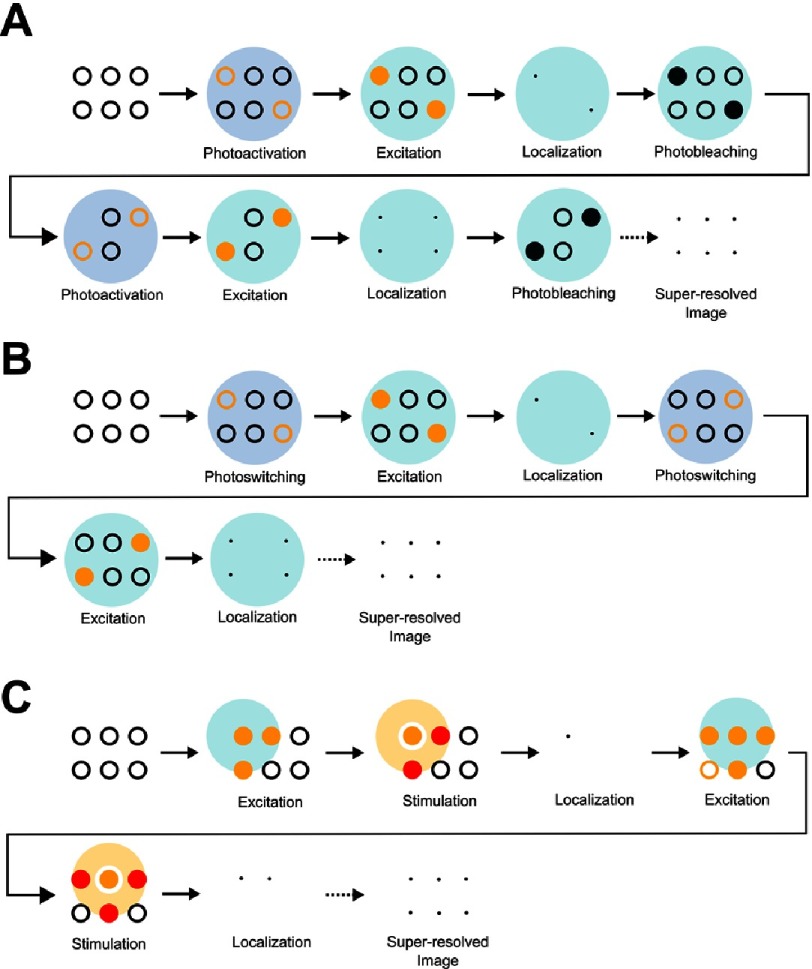
Schematic diagrams demonstrating the mechanism of operation of PALM, STORM and STED. In PALM (A), a randomly selected subset of a large number of closely-located inactive fluorophores (empty black circles) is activated using a weak short wavelength laser beam. The activated fluorophores (empty orange circles) are excited using a longer wavelength laser beam and the fluorescence (solid orange circles) is collected. Since the distance between the exclusively excited fluorophores is larger than the diffraction limit, they can be localized (black dots). The fluorophores are photo bleached under continuous excitation and another set of fluorophores is activated, excited, localized and photo bleached. This process is repeated until a complete super resolved image is obtained. In STORM (B), a randomly selected subset of a large number of closely located inactive switchable fluorophores (empty black circles) is switched using a weak short wavelength laser beam. The activated fluorophores (empty orange circles) are excited using a longer wavelength laser beam and the fluorescence (solid orange circles) is collected and localized. Instead of undergoing photo bleaching, the activated fluorophores are deactivated, or switched off, to a dark state by the application of a weak short wavelength laser beam, which in turn randomly activates (switches on) another subset of inactivated fluorophores. This is repeated until a complete super resolved image is obtained. In STED (C) a laser beam (large blue circle) excites a large number of active fluorophores (orange solid circles) in a diffraction limited region. A doughnut shaped beam (large orange circle), centered on the center of the excitation beam, de-excites all the excited fluorophores (red solid circles) except for the very few which are located at the center (orange solid circles). The stimulation of the de-excited fluorophores results in the emission of photons having a wavelength longer than that emitted spontaneously from the center of the doughnut shaped beam. Both emissions are filtered and the fluorophores at the center of the beam are localized (black dots) accordingly. By scanning the beam and repeating the excitation and stimulation processes, a complete super-resolved image is obtained.

Following localization, the active fluorophores are photobleached and another subset of inactive fluorophores is activated, excited, localized and photobleached. To ensure the localization of the majority of the fluorophores residing in the desired optical section, the aforementioned steps are repeated and the images of the localized fluorophores are overlapped until a satisfactory complete super-resolved image is produced (Figure 9A).

Since multiple fluorophores can be localized at the same time, PALM is considered to be a wide-field imaging technique. Although acquisition rates in wide-field imaging techniques (e.g. LSFM and TIRF) are typically considered to be limited by the detector’s frame rate, acquisition in PALM is relatively slow; limited by the total number of frames needed to acquire a complete super-resolved image. The affordable engineering of photoactivatable fluorophores and the applicability of PALM in TIRF and confocal (fPALM)^38^ configurations have encouraged its use in visualizing the dynamic architecture of the endocytic machinery^1^, and, the nanoscopic oligomerization of hemagglutinin in biological membranes^39^.

#### 4.2.2 Stochastic Optical Reconstruction Microscopy (STORM)

**Figure 10.**
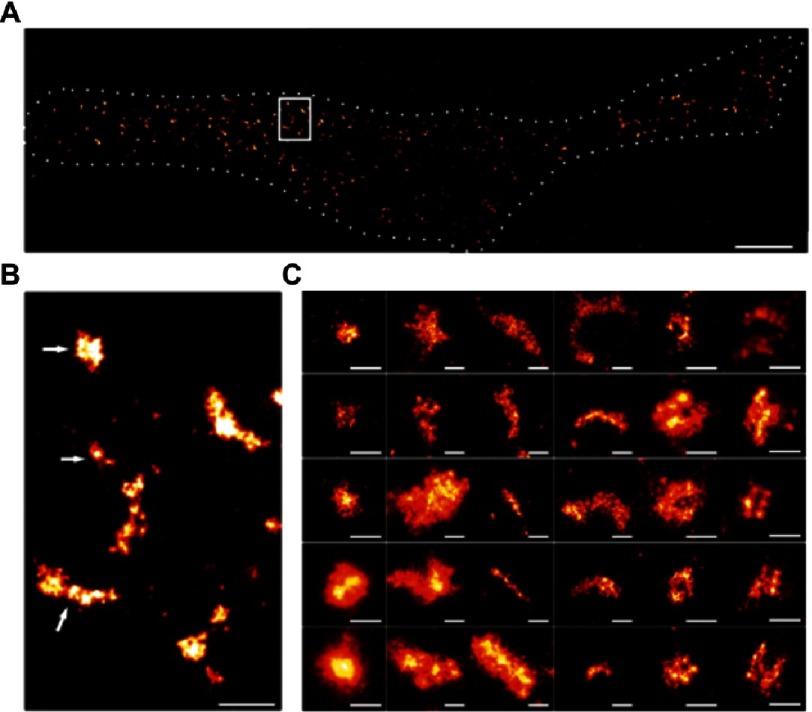
Apoptosis, a form of programmed cell death, is fundamental to the development of multi-cellular organisms. BAX, a pore-forming protein and key player in the cell suicide program, promotes apoptosis by irreversibly permeabilizing the mitochondrial outer membrane. The organization of BAX pores is unresolvable using diffraction-limited microscopy techniques. (A) AF647-anti-GFP nanobodies-labeled BAX in the outer membrane of apoptotic mitochondria (dashed white line) of HeLa cells imaged using dSTORM; a derivative of STORM that eliminates the requirement for using an activation laser by embedding the specimen in ’blinking buffer’^65^. Scale bar = 5 µm. (B) enlarged region of (A) showing non-random structures. Scale bar = 500 nm. (C) Gallery of apoptotic pores forming varied structures: dots, aggregates, lines, arcs, rings and double lines. Scale bar = 100 nm. Reproduced with permission from [43].

**Figure 11.**
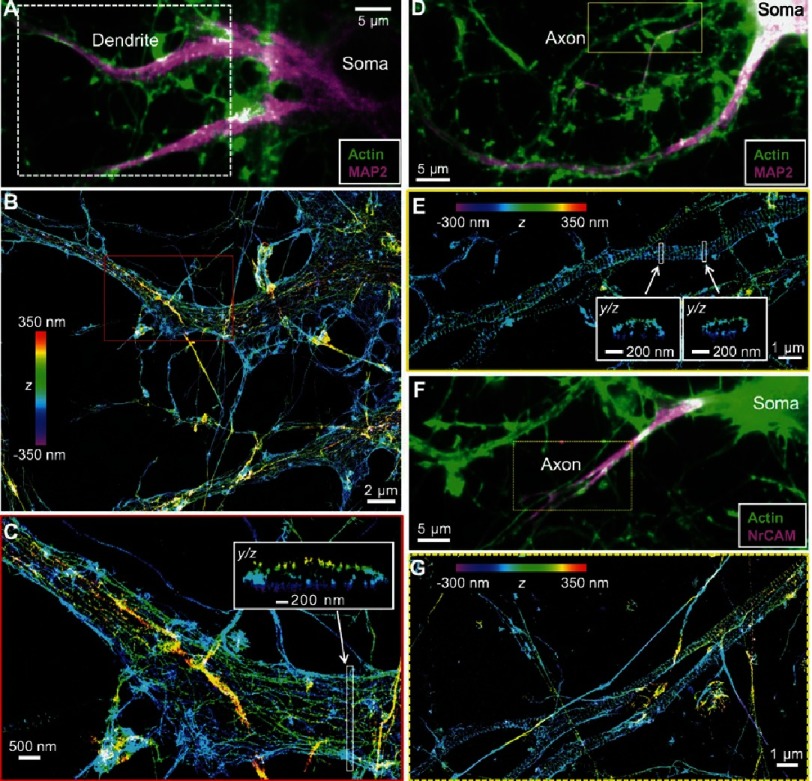
Spatial organization of actin in neuronal axons and dendrites imaged using STORM. (A, F) Diffraction-limited images of Alexa-Fluor647-labeled actin filaments in hippocampal neurons. (B, C, E, G) Super-resolved enlargements of the areas marked in (A, B, D, F) revealing the periodic distribution of actin in neurons with a uniform spacing of 180 –190 nm. Reproduced with permission from [44].

The photobleaching of active fluorophores, in PALM, has been traditionally seen as an additional time consuming step, which if eliminated, would enable faster imaging. PALM and STORM share common operation principles, in particular, the random activation and localization of a subset of fluorophores. Nonetheless, the use of STORM is distinguished by the use of photoswitchable fluorophores which can be activated (turned on) and deactivated (turned off) at will (Figure 9B)^40^. This improvement discards the requirement for successively photobleaching and activating fluorophores, thereby shortening the total acquisition time. STORM was first demonstrated using single fluorophores, however, the use of fluorophore-pairs was recently reported^41,42^ where, the emission of an activator fluorophore, following excitation, was shown to activate a photoswitchable reporter fluorophore; this process, when iterated, yields super-resolved images. STORM has been recently used in elucidating the mechanism of actions of the Nucleoid-associated proteins in chromosome organization^11^, visualizing the architecture of apoptotic pores^43^ (Figure 10), and, the periodic arrangement of actin in axons^44^ (Figure 11).

#### 4.2.3 Ground State Depletion followed by Individual return Microscopy (GSDIM)

**Figure 12.**
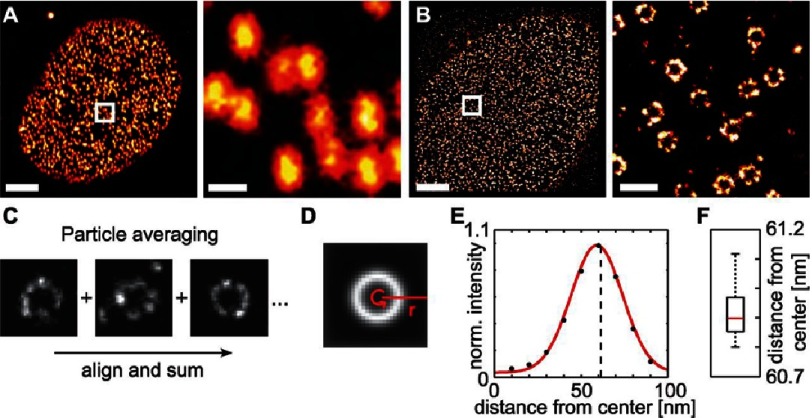
Structure of the Nuclear Pore Complex (NPC) analyzed by super resolution microscopy and particle averaging. GFP-tagged nucleoporins (NUPs) in the nuclear apical membrane of U2OS cells imaged using (A) confocal microscopy, and, (B) GSDIM. Scale bars = 1 µm (left), 300 nm (right). (C) Alignment and superposition of thousands of super-resolved NUPs results in a (D) circular form whose (E, F) average radius can be easily deduced. By labeling individual NUPs at different sites and measuring the radius of the computed circle, the alignment of the NUPs in individual NPCs can be deduced. Reproduced with permission from [8].

GSDIM relies in its operation on depleting the ground state by exciting all fluorophores in a field of view to an upper state^45,46^. The excited electrons relax to a long-lived dark “off” state by irradiative emission. Individual molecules return back, stochastically, from the dark state to the ground state by emitting a photon. The stochastically-emitted fluorescence is localized and a complete super-resolved image of a sample is constructed following the return, and localization, of all the excited fluorophores. Similar to other super resolution microscopy techniques, GSDIM has been instrumental in facilitating numerous discoveries as, for instance, visualizing the structural organization of the nuclear pore complex^8^ (Figure 12).

#### 4.2.4 Stimulated Emission by Depletion (STED) microscopy

As opposed to stochastic functional techniques like STORM and PALM, where a random subset of fluorophores is imaged at any one time, STED is a spatially-targeted technique denoting that a few fluorophores at a certain position are chosen to be imaged at any one time. STED exploits the difference in the wavelength between spontaneous and stimulated emission^47^. A collimated beam excites a large number of fluorophores in an optical section and another, doughnut shaped beam, of larger wavelength, centered at the excitation beam forcefully depresses the majority of the fluorophores to the ground state. The size of the annulus of the doughnut shaped beam can be adjusted down to molecular scales. The fluorophores in the middle of the doughnut-shaped beam, emit spontaneously whilst the forcefully depleted fluorophores emit stimulatingly.

Since both emissions possess different wavelengths, they can be separated and the spontaneously emitted fluorescence signal is localized. Scanning the excitation, and stimulation, beams laterally across the sample yields a complete super-resolved image of the optical section (Figure 9C). It can be shown that the obtained resolution using STED is inversely proportional to the intensity of the doughnut shaped beam. Therefore, the use of STED requires the engineering of special fluorophores^48^ that can withstand high laser powers and that the entire sample is not damaged at such high laser intensities.

**Figure 13.**
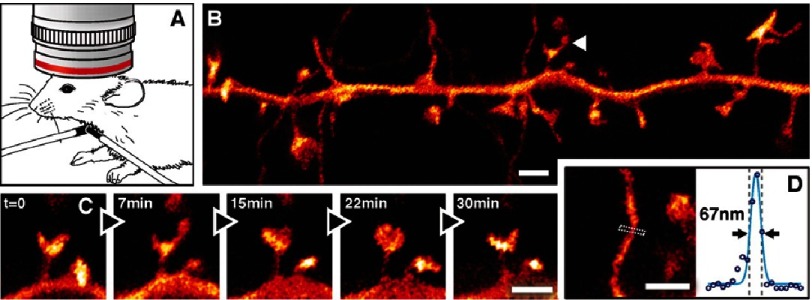
STED nanoscopy of the somatosensory cortex in the brain of a living mouse. (A) Optical access to the cortex of anesthetized YFP-expressing heterozygous mice is facilitated by the presence of a cover glass inserted through a sealed hole in the skull of the mouse. (B) Super-resolved projection of neuronal structures revealing (C) temporal dynamics of spine morphology. (D) Measurement of the width of a spine cross-section reveals a four-fold resolution improvement compared to diffraction-limited microscopy. Scale bar = 1 µm. Reproduced with permission from [50].

**Figure 14.**
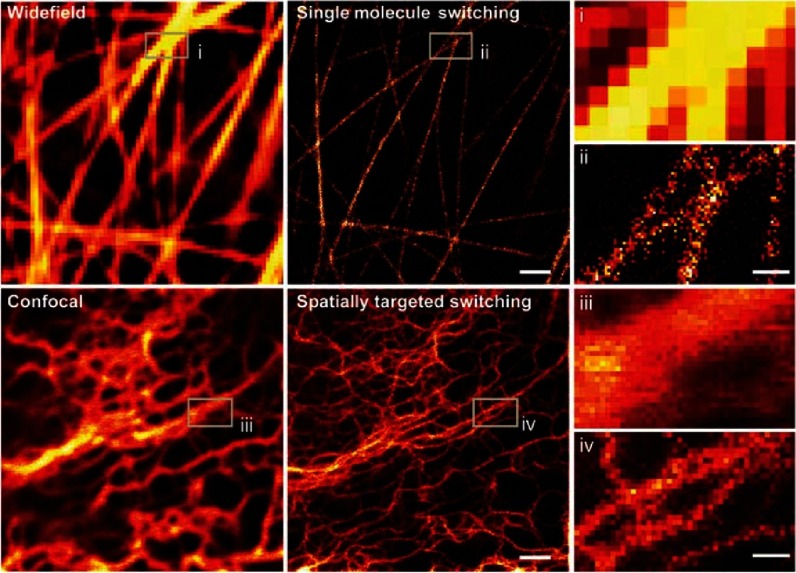
Living ptk2 cells expressing the fluorescent dye Dreiklang-Map2 imaged using: (i) Epi-fluorescence microscopy, (ii) STORM, (iii) confocal microscopy, and, (iv) STED microscopy. Scale bar = 1 µm. Reproduced with permission from [66].

STED has been used in the study of a number of biological systems including the clustering of the envelope protein during the budding of HIV-1 particles^7^, the periodic organization of neural cytoskeleton^6^ and the transient formation of cholesterol-mediated nanoscopic complexes in biological membranes^49^. STED is the first super resolution microscopy technique to be successfully tested on living animals^50^ (Figure 13). Images obtained using diffraction-limited and super-resolution microscopies are shown in Figure 14.

#### 4.2.5 Structured Illumination Microscopy (SIM)

As opposed to STED, where the true likeness of a sample is obtained after imaging, SIM is another super resolution technique that exploits the ability of periodic illumination to produce negative imprints of a sample. SIM employs a grid-like light pattern to illuminate a sample^51–53^. The illumination pattern is periodic with high intensity regions a wavelength-separated from low intensity regions. The high intensity regions excite the underlying fluorophores leaving the fluorophores underlying the low intensity regions in the ground state. By rotating the illumination pattern, an image of dark regions on a bright background (negative imprint) can be obtained.

**Figure 15.**
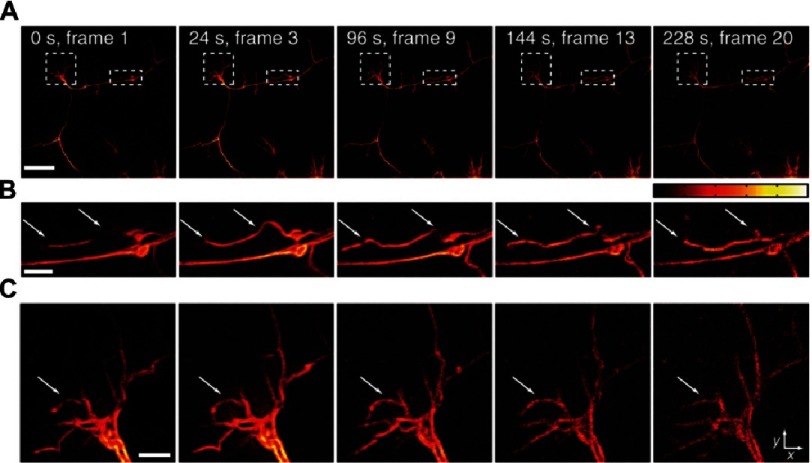
(A) Time series of filopodia-like structures expressing the fluorescent protein Lifeact-Dronpa imaged using parallelized STED nanoscopy. Scale bar = 10 µm. (B, C) Enlargements of the regions marked in (A). Scale bar = 2 µm. Reproduced with permission from [54].

To obtain a true super-resolved image, the negative imprints undergo advanced image processing, therefore, SIM requires that the sample be immobilized during pattern rotation and on other hand, non-trivial computation expertise to deconvolve the obtained images. The speed of acquisition is fast but is generally limited by the speed of pattern rotation and the total number of images acquired during this process. Recently, however, SIM’s pattern illumination scheme has inspired the development of a 100,000 folds faster variant of STED microscopy^54^. The reported scheme relies on orthogonal illumination obtained by passing two STED beams across two perpendicularly aligned diffraction gratings that split the beams onto periodic positions across the sample plane. The recently developed technique has been demonstrated on the imaging of 100-squared microns of GFP-labeled Keratin at less than a second temporal resolution and the dynamics of filopodia-like structures at relevant time scales (Figure 15).

#### 4.2.6 Binding-Activation Localization Microscopy (BALM)

**Figure 16.**
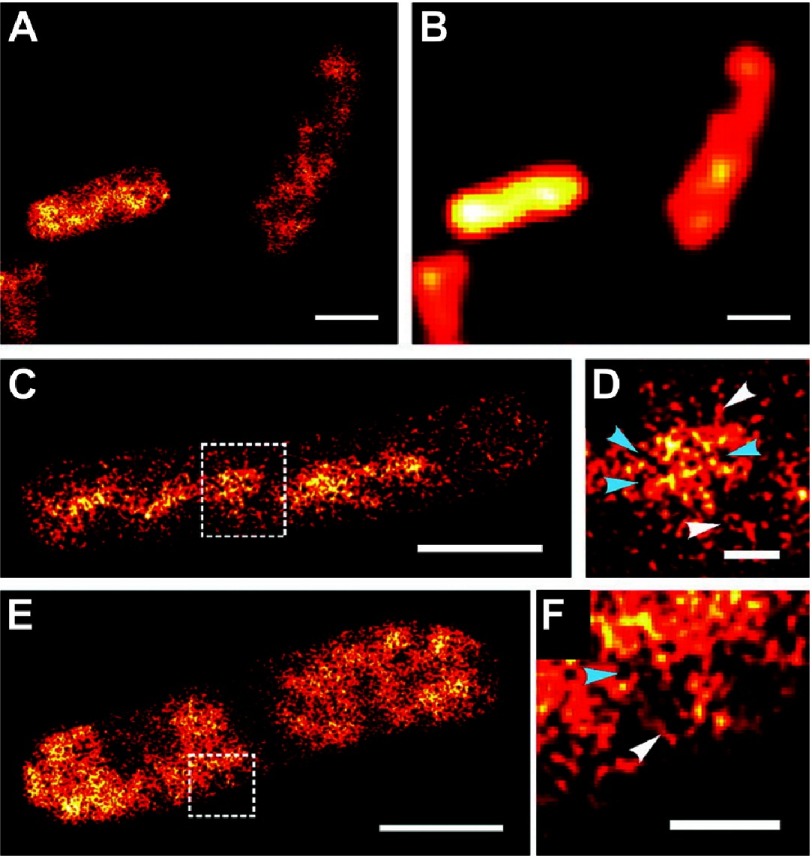
PicoGreen-labeled nucleoid structures in *E.Coli* imaged using (A) BALM and (B) diffraction-limited microscopy. (C, E) Spatiotemporal organization of nucleoids during bacterial division. (D, F) are enlarged regions of (C, E) showing void regions. Scale bars = 1 µm (A - C, E), 200 nm (D, F). Reproduced with permission from [55].

Dissimilar to PALM, STORM, STED and SSIM which can only provide positional information, BALM can also provide binding kinetics. BALM was developed with the aim of visualizing the organization of the bacterial chromosomes in E coli^55^. The DNA binding dyes, YOYO-1 and PicoGreen, were used to visualize the organization of DNA strands at a resolution of 10 nm (Figure 16). The fluorescence of the DNA-binding dyes increases up to 800-fold upon binding. A complete super-resolved image of the sample under study is obtained by the repeated localization and photobleaching of the sequentially binding fluorophores. BALM was also used to visualize the structural organization of amyloid fibrils^9^.

#### 4.2.7 Scanning Near field Optical Microscopy (SNOM)

SNOM was developed to break the diffraction limit of light by abolishing the far field, lens-based, imaging system using a tip brought close to a sample, in the near field, to allow imaging at sub-diffraction limited resolutions^56^. SNOM is commonly implemented in a TIRF configuration, whereby the evanescent field resulting from the total internal reflection of an incident beam can be detected and imaged using a fiber probe placed at a distance less than its penetration depth. The use of a scanning probe to construct a super resolved image of a sample implies that the acquisition rates using SNOM are limited by the probe scanning speed and that the sample, and probe, needs to be stabilized for good quality imaging. These requirements limit the use of SNOM in biological investigations.

## 5. Concluding remarks

The choice of a microscope is dependent on the spatial organization of the biological entity under study, its temporal dynamics and susceptibility to phototoxicity (Table 2). Generally, super-resolution microscopy techniques offer high spatial resolutions at the expense of slow acquisition rates. This rule does not apply for parallelized STED which affords super resolution imaging at high temporal resolutions but with toxic high light levels.

**Table 2:** Comparison between different fluorescence microscopy techniques.

Type	Imaging mode	Technique	Principle of operation	Spatial / temporal resolutions^**^	Compatible fluorescent label(s)^*^	Limitations	Compatible samples	Applications	References
Diffraction-limited	Wide field	**TIRF-M**	Spatial confinement of emission by exciting at an angle above the critical angle	200 nm / 2 ms	All	Diffraction-limited resolution	Plasma membrane and substrate supported artificial lipid bilayers	Imaging and tracking of single protein molecules in cellular and reconstituted membranes	^24,26,27,74,75^
		**LSFM**	Excitation using a periodic structure of light sheets followed by orthogonal collection of emission to construct an image	200 nm / 2 ms	All	Diffraction-limited resolution	*Drosophila melanogaster*, HeLa, *T. thermophile*, HL-60, and, *Caenorhabditis elegans* cells	Imaging binding kinetics of transcription factors, organization of nuclear lamins, microtubule movement during mitosis, and, Polymerase organization	^10,33,34^
	Scanning	**Confocal**	Spatial confinement of excitation / emission to a small volume followed by lateral sample / laser scanning to construct an image	200 nm / 5 ms for 512 pixels × 512 pixels	All	Diffraction-limited resolution and Slow acquisition rates	For a list of reported samples, refer to [76]	For a list of reported applications, refer to [76]	^76,77^
Super resolution	Wide field	**PALM**	Stochastic (random) activation of different subsets of spatially distinct fluorophores followed by localization and photobleaching to construct a super-resolved image	20 nm / 1 min for 15 µm^2^	For a list of STORM-compatible fluorescent dyes refer to [78]	Slow acquisition rates	CHO, NIH 3T3, COS-7, FoLu and HFF-1 cells	Mapping bacterial Chemo taxis networks, imaging the interaction of adhesion complexes, dynamic clustering of Hemagglutinin, organization of gene clusters, and, bacterial cell division.	^37,38,79–81^
		**STORM**	Stochastic switching of different subsets of spatially distinct fluorophores followed by localization to construct a super-resolved image	9 nm / 25 min for 1500 µm^2^	For a list of STORM-compatible fluorescent dyes refer to [78]. Compatible with ZnS-coated CdSe Qdot705^82^	Slow acquisition rates	IMR90, Ganglion, *Drosophila*, BSC-1, Cos-7, Sperm Flagella and IgG1 cells and *in vitro*	Genetic profiling *in vivo*, imaging chromosomal organization *in vivo*, periodic cytoskeleton organization, viral entry, coating and budding, and, endocytosis	^2,3,40,41,83–86^
		**GSDIM**	Ground state depletion followed by stochastic return of single molecules and localization to achieve super resolution imaging	30 nm / 30 min for 50 µm^2^	Rhodamine 6G, ATTO 532, ATTO 565, EGFP, EYFP, Citrine and PhiYFP	High laser powers and slow acquisition rates	MDCK and PtK2 cells	Imaging Epithelial morphogenesis	^45,46,87^
		**SIM**	Illumination using a structured (periodic) pattern followed by sample or pattern rotation to achieve super resolution imaging	50 nm / 5 s for 225 µm^2^	All	High computational demand	*Drosophila melanogaster* S2, HeLa, U2OS, and, RPE cells	Imaging mitochondria, Clathrin-coated vesicles, and the actin cytoskeleton	^51–53^
		**BALM**	Dye activation upon binding to substrate followed by fluorescence localization to construct a super-resolved image	14 nm / 10 min for 100 µm^2^	YOYO-1, PicoGreen and LCO-pFTAA	Slow acquisition rates	*E. Coli* and *in vitro*	Imaging the organization of bacterial chromosomes and the structure of amyloid fibrils	^9,55^
	Scanning	**STED**	Stimulated emission of fluorophores by depletion followed by collection of spontaneous emission and scanning to construct a super-resolved image	20 nm at 3.8 mW / 1 ms for 10 µm^2^^***^	For a list of STED-compatible fluorescent dyes refer to [88]. Compatible with ZnS-coated CdSe Qdot705^89^	High laser powers and slow acquisition rates	Cardiac myocytes, *Rattus norvegicus*, U2OS, NRK fibroblasts, IA32 MEFs, *Drosophila*, HEK293 and PtK2 cells and substrate supported lipid bilayers	Mapping the dynamic organization of cellular membranes, imaging receptors’ clustering during synapsis, and, recruitment and sorting of signaling proteins	^4–7,47,49,54,90–94^
		**SNOM**	Excitation, or collection of emission, in the near field (close to the sample) to achieve super resolution	10 nm / 100 s for 65 µm^2^	All	Slow acquisition rates	Plasma, and nuclear, membranes of *Xenopus laevis*, T lymphoma, and, dendritic cells	Imaging the organization of cellular membranes, clustering of membrane receptors and transport dynamics at nuclear pores	^56,95–97^

**Notes.**

* Non-exhaustive

** Approximate value

*** Using parallelized STED beams

The use of high light levels to perform super resolution imaging has strongly hampered the progress of live-cell imaging. Combining super resolution microscopy techniques is a popular approach to mitigate this problem. For example, multiple off-state transitions (MOST), also known as protected STED, combines the working principle of stochastic localization microscopy techniques, such as STORM or PALM, with STED to offer 2D coordinate-targeted fluorescence nanoscopy with low light levels^57^.

**Figure 17.**
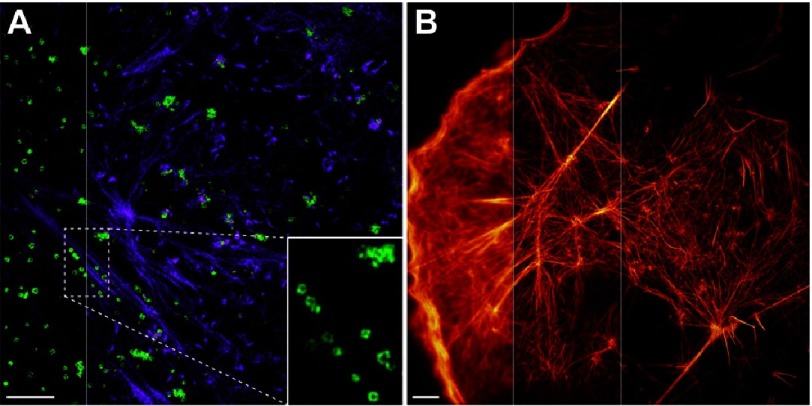
The evolution of endocytosis has never been resolved in real time. With the exception of SIM, all super resolution microscopy techniques are slow and intrinsically require the use of high light levels which are incompatible with imaging *in vivo*. Although SIM can resolve biological structures with relatively high temporal resolutions and low non-toxic light levels, its 100 nm spatial resolution could have not improved; rendering itself unsuitable for imaging sub-100 nm protein assemblies. To permit live-cell imaging at low non-toxic light levels, the spatial resolution of SIM can be improved by either exciting a sample at ultra-large incident angles (TIRF-SIM), or, coupling to a stochastic functional technique (STORM-SIM). Both techniques improve the spatial resolution of SIM without compromising the temporal resolution. (A) Endocytic (green) and cytoskeletal (blue) dynamics imaged using TIRF-SIM with an improved spatial resolution of 84 nm. Enlarged inset shows the growth of endocytic pits. Scale bar = 2 µm. (B) mApple-labeled actin filaments in COS-7 cells imaged using TIRF (left), TIRF-SIM (middle) and STORM-SIM (right) showing an improvement in resolution from 220 nm to 62 nm. Scale bar = 3 µm. Reproduced with permission from [58].

The combination of STORM, SIM and LSFM was also reported to exclusively allow 3D live-cell super-resolution imaging using non-toxic low light levels at biologically-relevant timescales^58^ (Figure 17). Despite these advances, an unfortunate fact remains that the combined fluorescence microscopy techniques are, as yet, incapable of accessing the wide spectrum of time and lengths scales on which biological processes occur. Simultaneous non-fluorescent- and fluorescent-based imaging tools can probe the extremes of those scales. Correlative Light and Electron Microscopy (CLEM) is one example in which the angstrom spatial resolution of electron tomography is combined with the intermediate temporal resolution of fluorescence microscopy to construct functional models for the dynamics of protein assemblies^59,60^.

Simultaneous label-free scattering and fluorescence imaging has also emerged as a powerful tool blending the micro-second temporal resolution of scattering microscopy with the molecular specificity of fluorescence microscopy to image highly dynamic membrane processes^61^. These, and more, developments, which represent a summit in biophotonic research, have formed a pathway that should lead to a satisfactory futuristic understanding of the operating principles of the tiny biological machineries, however, a comprehensive bottom-up molecular-based understanding of the functions performed by tissues and organs is still lacking. The next era should witness combined efforts to develop more advanced microscopy techniques capable of deep-tissue imaging for longer and larger, smaller and faster^62^, to dissect the network of interactions of the simple biological building blocks and elucidate its complex role in directing and regulating life.

## ABBREVIATIONS

BALM: Binding Activated Localization Microscopy
FRET: Forster Resonance Energy Transfer
GSDIM: Ground State Depletion followed by Individual return Microscopy
LSFM: Light Sheet Fluorescence Microscopy
SNOM: Scanning Nearfield Optical Microscopy
SIM: Structured Illumination Microscopy
STED: Stimulated Emission by Depletion
STORM: Stochastic Optical Reconstruction Microscopy
TIRF: Total Internal Reflection Fluorescence
